# Prediction of Apple Slices Drying Kinetic during Infrared-Assisted-Hot Air Drying by Deep Neural Networks

**DOI:** 10.3390/foods11213486

**Published:** 2022-11-02

**Authors:** Xiao Huang, Yongbin Li, Xiang Zhou, Jun Wang, Qian Zhang, Xuhai Yang, Lichun Zhu, Zhihua Geng

**Affiliations:** 1 College of Mechanical and Electrical Engineering, Shihezi University, Shihezi 832000, China; 2 Engineering Research Center for Production Mechanization of Oasis Special Economic Crop, Ministry of Education, Shihezi 832000, China; 3 College of Food Science and Engineering, Northwest A&F University, Xianyang 712100, China; 4 Xinjiang Production and Construction Corps Key Laboratory of Modern Agricultural Machinery, Shihezi 832000, China

**Keywords:** apple slices, phenols, flavonoids, color, drying, deep neural network

## Abstract

The effects of temperature, air velocity, and infrared radiation distances on the drying characteristics and quality of apple slices were investigated using infrared-assisted-hot air drying (IRAHAD). Drying temperature and air velocity had remarkable effects on the drying kinetics, color, total phenol content, total flavonoid content, and vitamin C content (VCC) of apple slices. Infrared radiation distance demonstrated similar results, other than for VCC and color. The shortest drying time was obtained at 70 °C, air velocity of 3 m/s and infrared radiation distance of 10 cm. A deep neural network (DNN) was developed, based on 4526 groups of apple slice drying data, and was applied to predict changes in moisture ratio (MR) and dry basis moisture content (DBMC) of apple slices during drying. DNN predicted that the coefficient of determination (*R*^2^) was 0.9975 and 1.0000, and the mean absolute error (MAE) was 0.001100 and 0.000127, for MR and DBMC, respectively. Furthermore, DNN obtained the highest *R*^2^ and lowest MAE values when compared with multilayer perceptron (MLP) and support vector regression (SVR). Therefore, DNN can provide new ideas for the rapid detection of apple moisture and guide apple processing in order to improve quality and intelligent control in the drying process.

## 1. Introduction

China is the world’s major producer and processor of apples, with a whopping 1.993 million hectares planted in 2020 and an annual production of approximately 44.07 million tons, accounting for approximately 57% of global production [1]. Apples are highly nutritious and rich in dietary fiber, vitamins, phenolic acids, flavonoids, and other nutritional and bioactive components that benefit cardiovascular disease, diabetes, cancer, blood pressure, lipids, inflammation, and hypercholesterolemia [2,3,4]. However, the high moisture content (MC) of fresh apples is susceptible to bacterial contamination during storage, preservation, and transportation, which causes spoilage and deterioration [5]. Consequently, the flavor and commercial value of apples are significantly affected by bacterial contamination. Therefore, most apples are processed into dried apple slices, with the premise of maintaining the original quality. During the drying process, most of the moisture in fruits and vegetables can be removed, which maximizes microbial growth inhibition while extending shelf life, reducing weight, and facilitating transportation [6].

IRAHAD is frequently used in fruit and vegetable processing because of its uncomplicated construction, low capital, and uniform heating [7]. Onwude et al. (2019) investigated the effect of IRAHAD on the physicochemical properties of sweet potatoes and discovered that using IRAHAD substantially reduces the drying time and unit energy consumption compared with hot air alone [8]. Furthermore, Jeevarathinam et al. (2021) found that IRAHAD could considerably retain nutrients, such as curcumin and starch, in turmeric slices compared with hot air alone [9]. Different drying conditions play an important role in the influence of the physicochemical properties of agricultural products after drying. Such conditions include the drying temperature [9], air velocity [10], relative humidity [11], and infrared radiation distance [12]. Appropriate drying conditions can improve the drying rate (DR), reduce the loss of nutrients, and better retain the color of agricultural products [13]. Therefore, the optimal drying conditions for each agricultural product are worth exploring.

The tremendous advancement of artificial intelligence in recent years has provided many opportunities in the field of food drying [14]. Many machine learning methods have been used for the prediction of the MC of food products [15], characterization of material microstructure [16], drying quality prediction [17], and selection of optimal drying conditions [18]. Liu et al. (2020) explored the effects of varying drying conditions on vacuum-pulse-dried kiwifruit slices and applied an artificial neural network (ANN) to predict the optimal drying conditions [19]. By comparing the performance of the traditional mathematical model and multilayer perceptron (MLP) model in predicting the MC of cylindrical quince slices during drying, Chasiotis et al. (2020) reported that the prediction accuracy of MLP models was higher than that of traditional mathematical models [15]. The relationship between the drying time and MC of apple slices was established by Bora et al. (2018) by extracting the red, green, and blue (RGB) values of apple slice photographs during drying to predict the MC [20]. An extreme learning machine (ELM) was applied to predict the moisture ratio (MR) variation during the drying of broccoli florets after pretreatment with negligible errors between the predicted and actual calculated results [21]. The multilayer perceptron (MLP) was applied by Voca et al. (2021) to predict the quality parameters of corn kernels, as well as the weight and MC remaining during drying; the experimental results demonstrated the generalization and nonlinear mapping capabilities of the MLP [22]. MLP employs a sigmoid function for neuron input and output, which may lead to “gradient disappearance” during the training process as the number of hidden layers increases. In comparison with MLP, deep neural networks (DNNs) can employ various activation functions (e.g., ReLU, Maxout, Swish) for input and output, which can effectively avoid “gradient disappearance”. Thus, DNNs have multiple network layers and can extract richer and more complex features than MLP [23,24]. Therefore, the DNN may predict MC with higher accuracy than MLP.

This study considered the influence of the kinetic and physicochemical properties of apple slices, dried by IRAHAD, under varying drying temperatures, air velocities, and infrared radiation distances. These factors have not been reported in detail in the literature; thus, an in-depth understanding of this topic is valuable. DNN was employed to predict the moisture change process of apple slices, which may provide new ideas for the rapid detection of MC in apple slices.

The main contributions of this study include: (1) the effects of varying drying temperatures, air velocities, and infrared radiation distances on the kinetics of IRAHAD apple slices; (2) the analysis of the influence of varying drying conditions on the quality of IRAHAD apple slices; and (3) the establishment of a prediction model for the kinetics of IRAHAD apple slices based on DNN. 

## 2. Materials and Methods

### 2.1. Materials

Fuji apples were purchased from Shihezi Luzhu Jiuding Wholesale Agricultural Products Market (Shihezi, China) with an initial MC of 85.56% ± 1.0% (wet basis). The apples were stored in a laboratory refrigerator (FCD-365HA, Qingdao Haier Special Electric Freezer Co., Ltd., Qingdao, China) at 4 °C for less than 3 days. Before the start of each experiment, an apple slice color protection solution was prepared (citric acid (CA) [1.0%] and ascorbic acid (AA) [2%]). The apples were sliced crosswise to a thickness of 3.5 ± 0.5 mm and soaked in the color protection mixture for 10 min to prevent browning due to prolonged exposure to air. The surfaces of the apple slices were then blotted dry.

### 2.2. Integrated Infrared and Hot Air Drying

**Figure 1:**
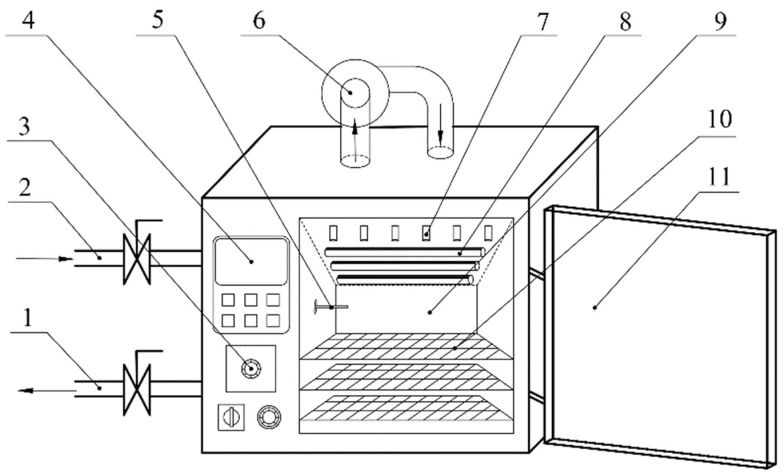
Principle diagram of IRAHAD dryer. 1. exhaust port, 2. intake port, 3. air velocity adjustment knob, 4. temperature control touch panel, 5. temperature transducer, 6. centrifugal fan, 7. air nozzle, 8. infrared heating tube, 9. drying chamber, 10. mesh drying tray support bracket, and 11. door. Reprinted from ref. [25].

IRAHAD experiments were conducted in an IRAHAD dryer (Taizhou Senttech Infrared Technology Co., Ltd., Taizhou, Jiangsu, China). The principle diagram of the dryer is shown in Figure 1. The temperature inside the dryer chamber was controlled to an accuracy of 0.1 °C. Air velocity inside the drying chamber was measured using an anemometer (AT816, SMART SENSOR, Thincol, Guangzhou, China) to an accuracy of 0.1 m/s. The dryer was run for 30 min before commencing the experiments, the machine was allowed to stabilize, and the corresponding conditions reached relative stability before the experiments were performed. The drying experiments were carried out at air temperatures of 50, 60, and 70 °C; air velocities of 1, 2, and 3 m/s; and infrared radiation distances of 10, 14, and 18 cm (Table 1). For each experiment, two trays of apple slices were placed in the drying chamber for drying, and the mass of each weight was 100 ± 1.0 g. Every 10 min, the trays were removed, and the apple slice samples were weighed using an electronic balance (BSM-4200.2, Shanghai Joujing Electronic Technology Co., Ltd., Shanghai, China) to an accuracy of 0.01 g.

### 2.3. Color Measurement

The color of the apple slices was measured using a colorimeter (CR400/410, Konica Minolta, Shanghai, China). The colorimeter was first calibrated and then used to determine the color value of fresh and dried apple slices. All color measurements for each apple slice sample were performed in triplicate and averaged for calculation. Equation (1) was used to compute the overall color difference of the apple slices [26].

(1) $$\Delta E_{\mathrm{apple}}=\sqrt{{\left(L_{{\mathrm{apple}}_{0}}^{*}-L_{{\mathrm{apple}}_{1}}^{*}\right)}^{2}+{\left(a_{{\mathrm{apple}}_{0}}^{*}-a_{{\mathrm{apple}}_{1}}^{*}\right)}^{2}+{\left(b_{{\mathrm{apple}}_{0}}^{*}-b_{{\mathrm{apple}}_{1}}^{*}\right)}^{2}}$$

where $\Delta E_{\mathrm{apple}}$ is the overall color difference value; $L_{{\mathrm{apple}}_{0}}^{*}$, $a_{{\mathrm{apple}}_{0}}^{*}$, and $b_{{\mathrm{apple}}_{0}}^{*}$ are the brightness, redness/greenness, and yellowness/blueness values of fresh apple slices, respectively; and $L_{{\mathrm{apple}}_{1}}^{*}$, $a_{{\mathrm{apple}}_{1}}^{*}$, and $b_{{\mathrm{apple}}_{1}}^{*}$ are the brightness, redness/greenness, and yellowness/blueness values of dried apple slices, respectively.

### 2.4. Measurement of Vitamin C

The vitamin C content (VCC) of the apple samples was determined by 2,6-dichlorophenol-indophenol (0.01 g/100 g solution) titration [27]. Dried apple samples were weighed on an analytical balance (BSA-120.4, Shanghai Joujing Electronic Technology Co., Ltd., Shanghai, China) to an accuracy of 0.1 mg, crushed, placed in a 100 mL volumetric flask, diluted to a total volume of 100 mL with an extraction solution (20 g/L oxalic acid solution), extracted for 15 min with an ultrasonic wave (KQ52000DE, Kunshan Ultrasonic Instruments Co., Shanghai, China), and then filtered. The filtrate (10 mL) was titrated with 2,6-dichlorophenol indophenol (0.01 g/100 g solution) until the solution became slightly red and did not fade after 15 s. In addition, 10 mL of a 20 g/L oxalic acid solution was used for blank titration. In this study, a comparative assessment of the VCC in apple samples under different drying conditions was performed, and all samples were analyzed in triplicate.

### 2.5. Measurement of Total Phenol Content (TPC) and Total Flavonoid Content (TFC)

The colorimetric method of Folin–Ciocalteu phenol reagent was used to determine the TPC of dried apple slices with slight modifications [28]. TPC extraction of dried apple slices was performed as follows: 2 g of dried apple samples were weighed, crushed, dissolved in 80% methanol solution, fixed to 30 mL, and sonicated for 30 min at 30 °C and 200 W. Following sonication, the extracts were placed in the laboratory refrigerator for 12 h at 4 °C. After refrigeration, the samples were placed in a benchtop high-speed centrifuge (LO-LX-M1850, Shanghai Lichen Bonsi Instrument Technology Co., Ltd., Shanghai, China) for 20 min at 8000 rpm, and filtered. The TPC was extracted twice.

The TPC of the apple slices was determined by firstly adding 0.4 mL of the total phenolic extract into a test tube, 2 mL of Folin–Ciocalteu solution, and 3 mL of sodium carbonate solution (20%). The resulting combination was allowed to react for 1 h under dark conditions. The absorbance of the reaction mixture was determined at 765 nm (UV-1900i, Shimadzu Instruments Co., Ltd., Suzhou, Jiangsu, China), and a standard curve was plotted using gallic acid standards as reference.

The TFC was measured using a UV spectrophotometer [29]. The total phenolic extract (1.5 mL) was placed in a test tube, and 1.5 mL of 5% sodium nitrite solution was added. After standing for 5 min, 150 μL of 10% aluminum chloride solution and 1 mL of 1 mol/L sodium hydroxide solution were added. The absorbance of the solution was determined at 510 nm using a UV spectrophotometer, and a standard curve was plotted using rutin standards as reference.

### 2.6. Drying Kinetic

The kinetic model for drying describes the MC parameters of the material, including the DBMC, MR, and DR. The MR of the apple slices can be calculated using Equation (2).

(2) $$MR^{\mathrm{apple}}=\frac{M_{t}^{\mathrm{apple}}-M_{e}^{\mathrm{apple}}}{M_{0}^{\mathrm{apple}}-M_{e}^{\mathrm{apple}}}$$

where $MR^{\mathrm{apple}}$ is the MR of the apple slices, $M_{t}^{\mathrm{apple}}$ is the DBMC of the apple slices at time *t*, $M_{e}^{\mathrm{apple}}$ is the DBMC of the apple slices when they are dried to equilibrium, and $M_{0}^{\mathrm{apple}}$ is the initial DBMC. The DBMC of the apple slices can be calculated using Equation (3).

(3) $$M_{t}^{\mathrm{apple}}=\frac{W_{t}^{\mathrm{apple}}-G^{\mathrm{apple}}}{G^{\mathrm{apple}}}$$

where $W_{t}^{\mathrm{apple}}$ is the weight of the apple slice at moment *t* and $G^{\mathrm{apple}}$ is the weight of the dry matter of the apple. DR can be calculated using Equation (4).

(4) $$N^{\mathrm{apple}}=\frac{M_{t_{1}}^{\mathrm{apple}}-M_{t_{2}}^{\mathrm{apple}}}{t_{1}-t_{2}}$$

where $N^{\mathrm{apple}}$ is the DR, $M_{t_{1}}^{\mathrm{apple}}$ is the DBMC at $t_{1}$, $M_{t_{2}}^{\mathrm{apple}}$ is the DBMC at time $t_{2}$, $t_{1}$ and $t_{2}$ are the two different drying times during the drying process.

### 2.7. Deep Neural Networks

#### 2.7.1. Structure of Deep Neural Network

**Figure 2:**
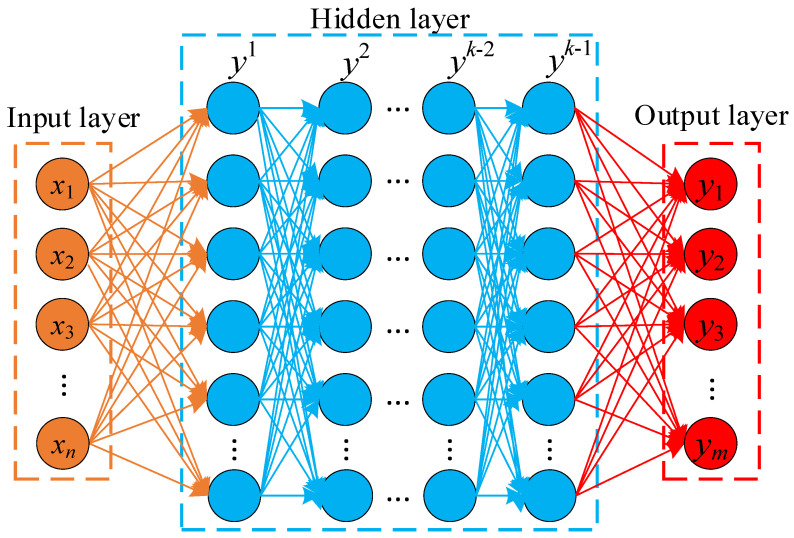
Structure of DNN.

A DNN is a feed-forward neural network that updates parameters, such as network weights and biases, via error back-propagation [30]. The network structure of a DNN typically consists of three parts (Figure 2) [31]. The input layer is used to input known features related to the training target. The hidden layer is typically composed of multiple layers of neurons stacked on top of each other with only adjacent layers of connected neurons. The output layer is employed to output the training target [32]. Data are passed between the input layer, each layer of the hidden layer, and the output layer utilizing an activation function, with the output of the previous layer used as the input to the next layer, as shown in Equation (5).

(5) $$y^{k}=\sigma (w^{k}y^{k-1}+b^{k})$$

where *k* is the number of layers of the DNN, σ(g) is the activation function, $w^{k}$ is the weight matrix of the connections between adjacent layers, $b^{k}$ is the bias matrix, and $y^{k}$ is the output of the layer *k*, which is calculated from the output $y^{k-1}$ of the previous layer.

#### 2.7.2. Modeling of Kinetic Curve Prediction

This section is based on the DNN and investigates 27 different types of IRAHAD apple slice datasets obtained from the experiments. A total of 4526 sets of apple slice sample data were obtained from the experiments. Of these, 70% (3627 sets) and 30% (899 sets) of the sample data were selected as the training set and test set, respectively.

**Figure 3:**
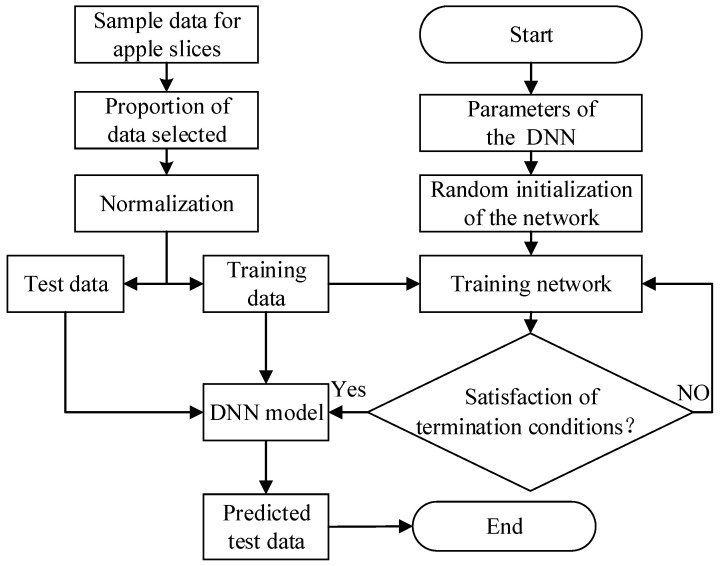
Flow chart of DNN model for predicting moisture content of apple slices.

Among all the drying data of the apple slices, the weights of the apple slices, drying time, drying temperature, drying air velocity, and infrared radiation distance were selected as the inputs of the DNN, and MR and DBMC were selected as the outputs. The main flow of the apple drying kinetic curve prediction model based on the DNN is shown (Figure 3).

(1) Raw data on the apple drying time, the weight of apple slices at different times, drying temperature, drying air velocity, and infrared radiation distance, were collected.
(2) The collated dry raw data were randomly selected as training samples and test samples in a certain proportion, and all sample data were normalized according to Equation (6).
  (6) $$x_{i}^{\mathrm{apple}}=a+(b-a)\frac{x^{\mathrm{apple}}-x_{\min}^{\mathrm{apple}}}{x_{\max}^{\mathrm{apple}}-x_{\min}^{\mathrm{apple}}}$$
  where a and b represent the upper and lower limits of the normalized range, respectively; $x^{\mathrm{apple}}$ represents the original data; $x_{\min}^{\mathrm{apple}}$ represents the minimum value of the sample data; $x_{\max}^{\mathrm{apple}}$ represents the maximum value of the sample data; and $x_{i}^{\mathrm{apple}}$ represents the normalized data.
(3) The DNN parameters were set and the normalized drying data were inputted.
(4) Training was started until a stopping condition was achieved.

In this study, the $R^{2}$ was applied to evaluate the degree of merit of the constructed model, [33] and the closer its value is to 1, the higher the accuracy of the prediction, and the closer the predicted value is to the true value. *R*^2^ can be obtained from Equation (7).

(7) $$R^{2}(y^{\mathrm{apple}},{\hat{y}}^{\mathrm{apple}})=1-\frac{\sum_{i=0}^{n}{\left(y_{i}^{\mathrm{apple}}-{\hat{y}}_{i}^{\mathrm{apple}}\right)}^{2}}{\sum_{i=0}^{n}{\left(y_{i}^{\mathrm{apple}}-{\bar{y}}_{i}^{\mathrm{apple}}\right)}^{2}}$$

In Equation (7), $y_{i}^{\mathrm{apple}}$ is the actual value, ${\hat{y}}_{i}^{\mathrm{apple}}$ is the predicted value, ${\bar{y}}_{i}^{\mathrm{apple}}$ is the average of actual values, and n is the number of training samples.

## 3. Results and Discussions

### 3.1. Effects of Different Drying Conditions on Drying Kinetic

**Figure 4:**
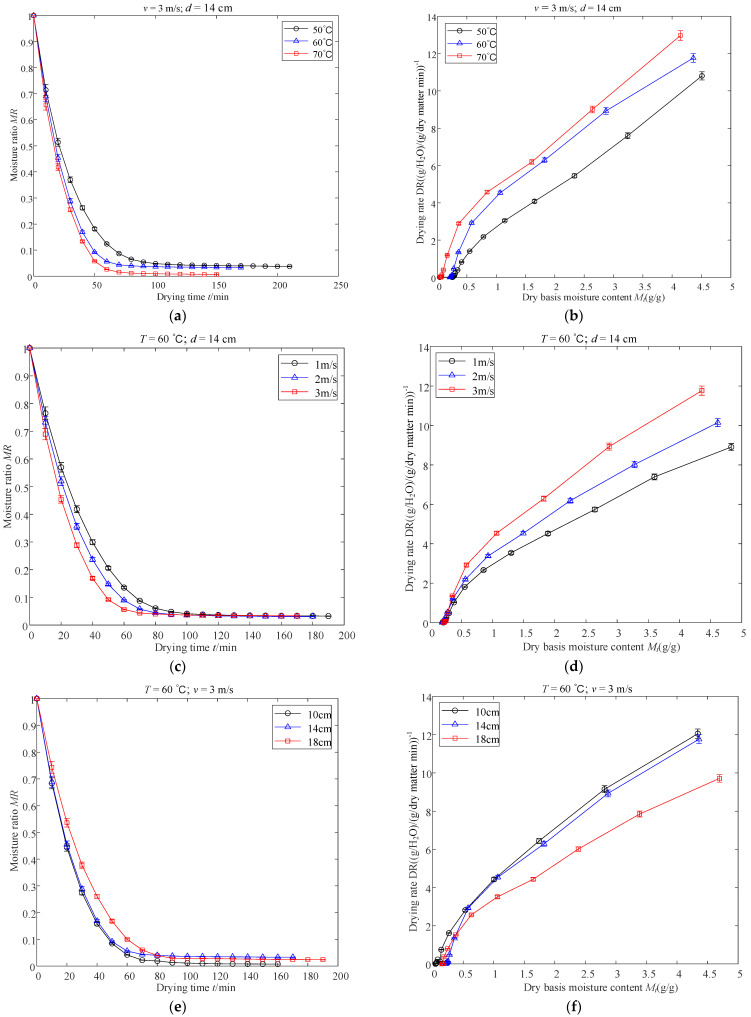
Drying kinetics (**a**,**c**,**e**) and drying rate (**b**,**d**,**f**) curves of apple slices at varying conditions.

The changes in MR versus time and DR versus DBMC for different drying temperatures, air velocities, and infrared radiation distances are shown in Figure 4, where *T* is the temperature, *v* is the air velocity, and *d* is the infrared radiation distance. As displayed in Figure 4a,b, the minimum drying time (150 min) was obtained when *T*, *v*, and *d* were set to 70 °C, 3 m/s, and 14 cm, respectively. The drying times of the apple slices at 60 °C and 50 °C were 170 min and 210 min, respectively. The drying time was reduced by approximately 13.33% and 40.00% for apple slices dried at 70 °C compared with those dried at 60 °C and 50 °C, respectively. Drying efficiency increased with increasing *T*. A possible reason for this phenomenon is that the heat transfer between the apple slice samples and the air inside the dryer was enhanced as the temperature increased, accelerating the diffusion of moisture inside the apple slices to the surface. Similar findings were consistent with the results obtained from kiwifruit slices and elephant cassava by Liu et al. (2020) [19] and Kosasih et al. (2020) [34], respectively.

As can be seen in Figure 4c,d, the drying time decreased from 190 to 170 min when *v* increased from 1 to 3 m/s with *T* = 60 °C and *d* = 14 cm. This clearly demonstrates that reducing *v* prolongs drying time. Similar conclusions were reported by Ye et al. (2021), who reported that increasing *v* in a drying chamber could enhance the drying efficiency of mint leaves [35]. This finding can be explained by the fact that convective heat transfer is enhanced by the increase in *v*, which accelerates the discharge of water vapor from the drying chamber [21].

Figure 4e,f shows the effect of *d* (10, 14, and 18 cm) on the drying curves of apple slices with a constant v of 3 m/s and a constant *T* of 60 °C. Additionally, the drying time of apple slices increased by 18.75% after increasing *d* from 10 cm to 18 cm. As expected, the farther *d* is, the more drying time the apple slices require during the drying process. The probable cause of this phenomenon is that the smaller the value of *d*, the greater the density of heat flux absorbed by the material, and the temperature inside the material increases, thereby accelerating the diffusion of water. This finding is consistent with the results obtained from ginger slices and green beans by Osae et al. [36] and Kaveh et al. [37], respectively.

### 3.2. Effects of Different Drying Conditions on TPC and TFC

**Figure 5:**
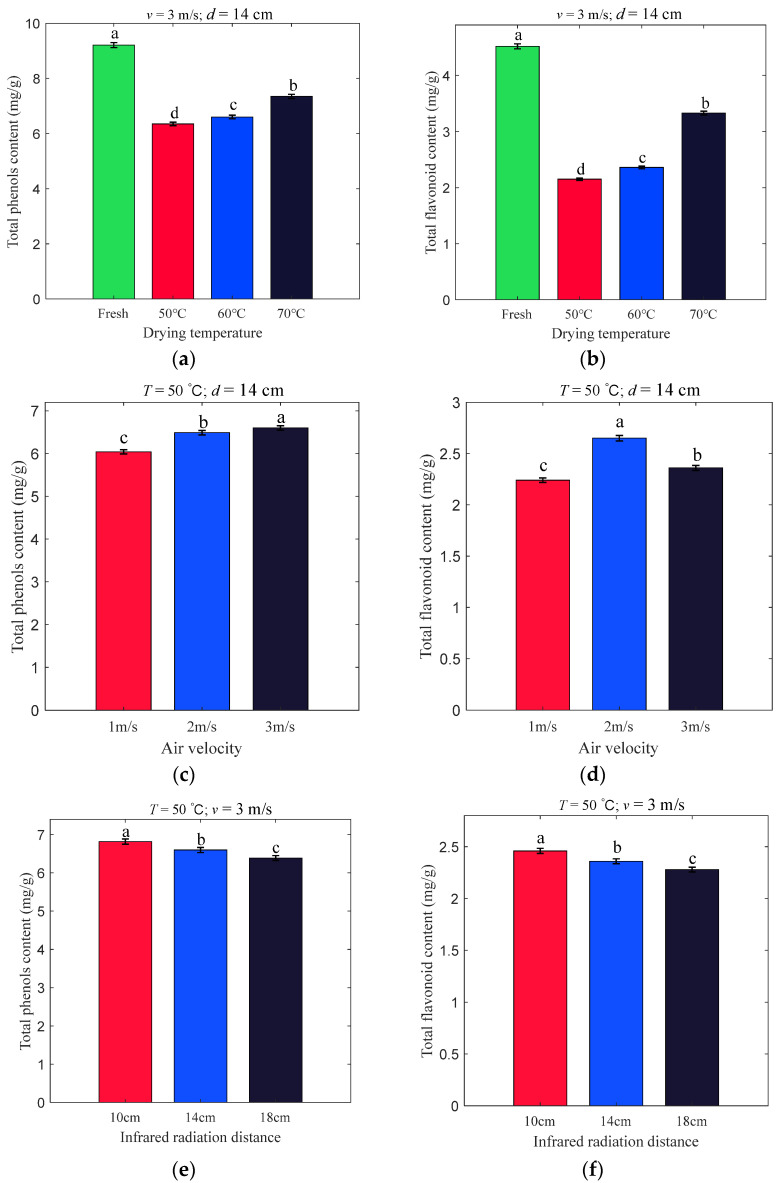
The TPC (**a**,**c**,**e**) and TFC (**b**,**d**,**f**) at varying drying conditions. Notes: In accordance with the Waller–Duncan test, different lowercase letters in the pictures revealed remarkable differences (*p* < 0.05).

The TPC and TFC of apple slices at different *T* (50, 60, 70 °C), *v* (1, 2, 3 m/s), and *d* (10, 14, 18 cm) are shown in Figure 5. The TPC and TFC of fresh apple slices were 9.21 mg/g and 4.52 mg/g, respectively. With *v* and *d* kept at 3 m/s and 14 cm, respectively, TPCs for *T* equal to 50, 60, and 70 °C were 6.35, 6.60, and 7.35 mg/g and TFCs were 2.15, 2.36, and 3.33 mg/g, respectively. When *T* was varied from low to high, the TPC and TFC retention tended to increase noticeably (TPC from 68.95% to 79.80% and TFC from 47.57% to 73.67%). A possible reason for this is that the least drying time is consumed at 70 °C, followed by 60 °C, which contributes to the reduction in the oxidation of phenolic and flavonoid compounds, as reported by Onwude et al. (2019) [8]. 

Figure 5c,d show that there is a notable tendency for TPC to increase as the *v* changes from weak to strong (6.04, 6.49, and 6.60 mg/g), and interestingly, TFC increases and then decreases (2.24, 2.65, and 2.36 mg/g). The increase in TPC can be explained by a stronger *v*, which causes a shorter drying time. The probable reasons for the variation in TFC can be analyzed as the drying time in the case of 2 m/s is shorter than that of 1 m/s. Meanwhile, in the case of 3 m/s, because of high air velocity, the oxygen exchange rate between the drying chamber and outside increases, accelerating the breakdown of the more oxidizable flavonoid compounds [38].

Figure 5e,f illustrate the continuous significant decrease in TPC and TFC due to the variation in *d* from 14 to 18 cm. This may be because the smaller the *d*, the shorter the time it takes to dry the apple slices, as described by An et al. [39] and Onwude et al. [8]. The time for browning and oxidation reactions is shortened, resulting in higher TPC and TFC.

### 3.3. Effects of Different Drying Conditions on Vitamin C

Vitamin C is an essential trace substance that participates in various biochemical reactions in the human body and plays a vital role in maintaining the growth of an organism [40]. However, vitamin C cannot be self-contained in the human body and must be supplemented by the intake of natural foods [19]. Fruits are an important source of vitamin C. Nevertheless, the factors affecting the stability of vitamin C include temperature, moisture, and oxygen [41]; hence, VCC is a commonly utilized indicator to evaluate nutrient retention during food processing [42]. 

**Figure 6:**
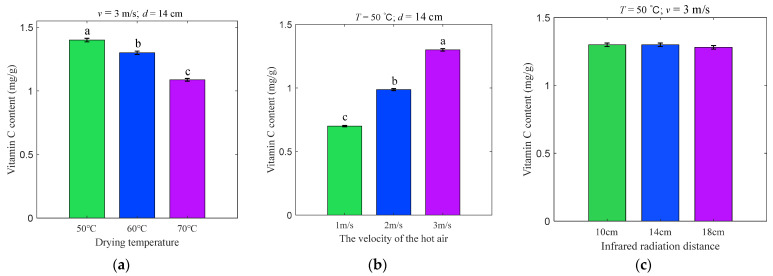
The VCC (**a**–**c**) at varying drying conditions. Notes: In accordance with the Waller–Duncan test, different lowercase letters in the pictures revealed remarkable differences (*p* < 0.05).

As displayed in Figure 6, both *T* and *v* have detrimental effects on VCC in apple slices, whereas *d* has no noteworthy effect on VCC. More precisely, the VCC of apple slice samples decreased in response to the enhancement of *T* and increased rapidly in response to the heightening of *v*. Similar findings were reported by Liu et al. [21], who studied broccoli florets and suggested that the occurrence of this phenomenon could be interpreted as a weakening of *T* and an increase in *v* reducing drying time, thus leading to the degradation of vitamin C. The maximum retention of VCC was obtained at *T*, *v*, and *d* equal to 50 °C, 3 m/s, and 14 cm, respectively.

### 3.4. Effects of Different Drying Conditions on Color

Color plays an important role in the quality of dried products and is related to their acceptability by consumers. The color parameters ($L_{\mathrm{apple}}^{*}$, $a_{\mathrm{apple}}^{*}$, and $b_{\mathrm{apple}}^{*}$) of the fresh and dried apple slices are listed in Table 2. It can be concluded that the IRAHAD of apple slices can improve the color of apple slices and increase the $L_{\mathrm{apple}}^{*}$, $a_{\mathrm{apple}}^{*}$, and $b_{\mathrm{apple}}^{*}$ of dried apple slices compared with fresh ones [43]. This phenomenon revealed that the drying method using IRAHAD could improve the brightness and yellowness and degrade the greenness (toward redness) of the dried apple slices. As shown in Table 2, the values of $a_{\mathrm{apple}}^{*}$ and $b_{\mathrm{apple}}^{*}$ of the dried apple slices exhibit a similar trend, whereas the values of $a_{\mathrm{apple}}^{*}$ and $L_{\mathrm{apple}}^{*}$ have the opposite trend. When other drying conditions were kept constant and the drying temperature was increased from 50 to 70 °C, the values of $a_{\mathrm{apple}}^{*}$ and $b_{\mathrm{apple}}^{*}$ decreased and then increased, which is likely because the VCC, which can be used as an anti-browning agent, is drastically reduced at 70 °C (Figure 4), increasing the enzymatic browning reaction [44]. This finding is consistent with Yao et al. [45] in their study of mango slices, and hence, the increase in both redness and yellowness. As the air velocity increased from 1 to 2 m/s, the values of $a_{\mathrm{apple}}^{*}$ and $b_{\mathrm{apple}}^{*}$ decreased significantly. This is likely attributable to the low VCC associated with 1 m/s treatment compared with 2 m/s, resulting in a decrease in both redness and yellowness. As shown in Table 2, the infrared radiation distance had no significant effect on the $L_{\mathrm{apple}}^{*}$ $a_{\mathrm{apple}}^{*}$ or $b_{\mathrm{apple}}^{*}$ of the dried apple slices. This is consistent with the results obtained by Zhang et al. (2020), who studied sponge-gourd slices [12].

The value of $\Delta E_{\mathrm{apple}}$ symbolizes the overall difference in color between fresh and dried products, and the closer it is to zero, the higher the color retention of the dried products [8]. The highest $\Delta E_{\mathrm{apple}}$ value was obtained at 60 °C with *v* = 2 m/s and *d* = 14 cm in Table 2. However, the highest $L_{\mathrm{apple}}^{*}$, $a_{\mathrm{apple}}^{*}$, and $b_{\mathrm{apple}}^{*}$ values occur at 60 °C with *v* = 2 m/s and *d* = 14 cm, 70 °C with *v* = 3 m/s and *d* = 14 cm, and 60 °C with *v* = 1 m/s and *d* = 14 cm, respectively. This discrepancy in color may be explained by a combination of factors such as enzymatic browning, vitamin C oxidation, and Maillard reactions [46,47].

### 3.5. Model Results and Analysis

The DNN in this study was constructed, computed, and run on a personal laptop (Windows 10, Intel i9-10885H, 2.4 GHz, RAM 128 GB) on MATLAB R2020b (9.9).

MC is the medium of a number of chemical reactions in the processing of fruits and is a vital quality factor in the measurement of processing; therefore, the speed of moisture loss greatly affects the quality of fruits. The variation in the MR and DBMC of the apple slices during drying is highly nonlinear. It is feasible to employ DNN modeling to represent this complex process, which can provide new ideas for the rapid detection of apple moisture and theoretical ideas to guide apple processing to improve quality as well as intelligent control in the drying process.

**Figure 7:**
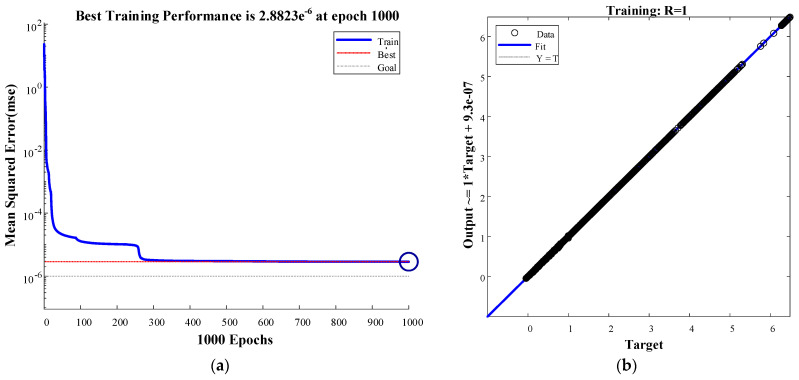
DNN training adaptation curve (**a**) and regression curve (**b**) based on apple slice drying data.

Figure 7a shows the training adaptation curve of the DNN. The solid blue line shows the mean square error (MSE) of the DNN, the red dashed line is the optimal training MSE, and the black dashed line represents the target MSE of the training. The MSE for each generation of the DNN training decreases almost linearly with values less than 10^−4^ until 50 iterations, after which its rate of decline tends to slow down and then decreases sharply again at approximately 270 iterations before leveling off and reaching a maximum value of 2.8823 × 10^−6^ for training at 1000 iterations. The regression curve plot for the DNN training is shown in Figure 7b. The circle indicates the training sample data, the solid blue line indicates the fitted curve, and the dashed black line indicates the target curve. The graph shows that the predicted value is almost consistent with the target value, with a correlation coefficient *of R* = 1.

**Figure 8:**
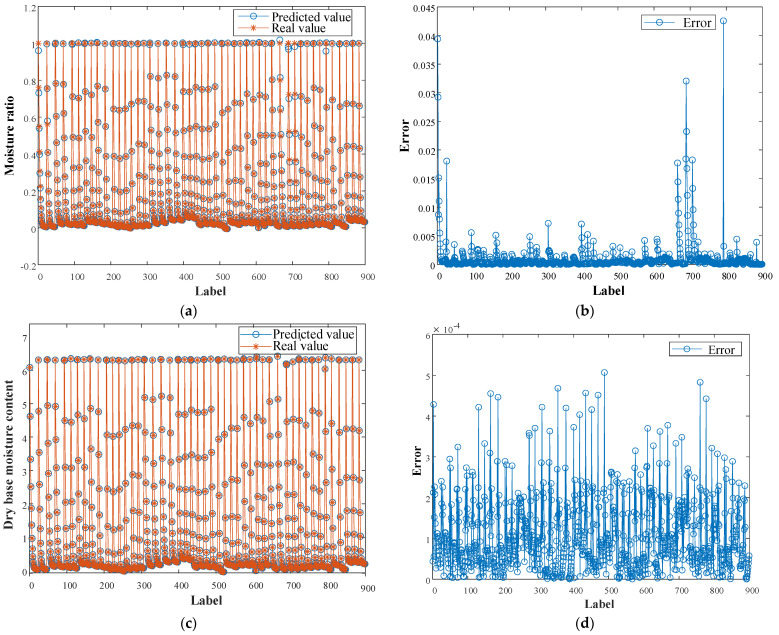
Predicted results (**a**,**c**) and error values (**b**,**d**) for MR and DBMC.

Furthermore, Figure 8 shows the prediction results of the MR and DBMC of the test set by the MC model of apple slices based on the DNN. Figure 8a shows the predicted MR results for the test set; the blue circles indicate the predicted values, and the red asterisks indicate the true values. The results show that only a small portion of the predicted values differ from the true values. Figure 8b shows the prediction error values for the MR of the test set, the maximum error value of the prediction is <0.045, and 90% of the predicted error values are <0.005. Figure 8c shows the predicted DBMC results for the test set. Figure 8d shows the predicted error values for the DBMC of the test set, with the maximum error value of the prediction being <0.0006. The prediction results show that the established DNN has high prediction accuracy for the MR and DBMC of apple slices and the prediction error values are in an acceptable range.

To verify the superiority of the developed model for predicting the MC of apple slices, MLP, SVR, and DNN were employed to predict the MR and DBMC, with the same training and test samples for the three methods. The *R*^2^ and MAE values of the results predicted by the three methods are listed in Table 3.

From Table 3, the calculated *R*^2^ values for MR and DBMC were 0.0015, 0.0042, and 0.0442, 0.1010 higher than MLP and SVR, respectively, and the predicted MR and DBMC calculated MAE values were 78% and 97.73% lower than MLP and 85.71% and 99.86% lower than SVR, respectively.

In summary, the MC prediction model for apple slices developed in this study has higher accuracy and better generalization performance than those developed by MLP and SVR. Therefore, the DNN can achieve MR and DBMC predictions for apple slices under varying drying conditions.

## 4. Conclusions

The results of this study show that varying drying conditions have different influences on the drying curve and quality of the dried apple slices. The conclusions are summarized as follows:

(1) The drying temperature, air velocity, and infrared radiation distance remarkably affected the drying rate of apple slices. The drying time of apple slices decreased with an increase in temperature and air velocity, and prolonged with an increase in infrared radiation distance.
(2) The drying temperature and air velocity had remarkable effects on the TPC, TFC, VCC, and color of the dried apple slices, while the infrared radiation distance had a significant effect on TPC and TFC. The TPC, TFC, and VCC decreased with increasing drying temperature. With increasing air velocity, TPC and VCC increased, and TFC increased at first and then decreased. An increase in infrared radiation distance decreased TPC and TFC but had no remarkable effect on VCC and color.
(3) A DNN prediction model for the MR and DBMC of apple slices was established based on the drying data of apple slices. The *R* value of the DNN was 1, and the *R*^2^ values of the MR and DBMC were 0.9975 and 1, respectively. Comparing the prediction results of the DNN model with those obtained by the SVR and MLP models, the *R*^2^ and MAE of the DNN model are better than those of the SVR and MLP, indicating that the DNN model has stable robustness.

## Figures and Tables

**Table 1:** The combinations of varying drying conditions.

Number	Drying Temperature (°C)	Air Velocity (m/s)	Infrared Radiation Distance (cm)
1	50	1	10
2	50	1	14
3	50	1	18
4	50	2	10
5	50	2	14
…	…	…	…
9	50	3	18
10	60	1	10
…	…	…	…
18	60	3	18
19	70	1	10
…	…	…	…
27	70	3	18

**Table 2:** Comparison of color parameters of apple slices at varying conditions.

Parameter	Fresh	Drying Temperature (*d* = 14 cm, *v* = 3 m/s)			Air Velocity (*T* = 60 °C, *d* = 14 cm)			Infrared Radiation Distance (*T* = 60 °C, *v* = 3 m/s)		
		50 °C	60 °C	70 °C	1 m/s	2 m/s	3 m/s	10 cm	14 cm	18 cm
	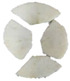	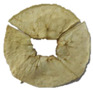	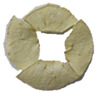	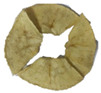	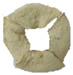	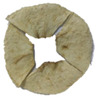	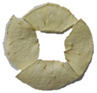	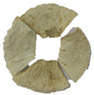	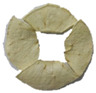	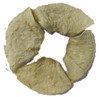
$L_{\mathrm{apple}}^{*}$	72.07 ± 0.45 ^d^	85.45 ± 0.29 ^b^	86.48 ± 0.26 ^a^	83.61 ± 0.23 ^c^	85.58 ± 0.07 ^c^	87.23 ± 0.25 ^a^	86.48 ± 0.26 ^b^	85.82 ± 1.00 ^a^	86.48 ± 0.26 ^a^	86.56 ± 0.64 ^a^
$a_{\mathrm{apple}}^{*}$	−2.96 ± 0.59 ^c^	0.99 ± 0.21 ^a^	0.04 ± 0.43 ^b^	1.74 ± 0.18 ^a^	1.06 ± 0.12 ^a^	0.24 ± 0.20 ^b^	0.04 ± 0.43 ^b^	0.80 ± 0.41 ^a^	0.04 ± 0.43 ^a^	−0.15 ± 0.59 ^a^
$b_{\mathrm{apple}}^{*}$	23.57 ± 1.22 ^c^	28.31 ± 1.05 ^b^	30.37 ± 0.63 ^a^	28.38 ± 1.13 ^a,b^	30.68 ± 1.00 ^a^	29.77 ± 1.05 ^a^	30.37 ± 0.63 ^a^	28.53 ± 0.88 ^a^	30.37 ± 0.63 ^a^	30.02 ± 1.29 ^a^
$\Delta E_{\mathrm{apple}}$	-	14.76 ± 0.60 ^b^	16.23 ± 0.25 ^a^	13.39 ± 0.52 ^c^	15.80 ± 0.48 ^b^	16.72 ± 0.28 ^a^	16.23 ± 0.25 ^ab^	15.13 ± 0.76 ^b^	16.23 ± 0.25 ^a^	16.16 ± 0.07 ^a^

Notes: In accordance with the Waller–Duncan test, different lowercase letters in the same row of the table revealed remarkable differences (*p* < 0.05).

**Table 3:** Test set *R*^2^ and MAE values for different methods.

Methods	MR		DBMC	
	*R* ^2^	MAE	*R* ^2^	MAE
DNN	0.9975	0.0011	1.0000	0.000127
MLP	0.9960	0.0050	0.9958	0.005600
SVR	0.9533	0.0077	0.8990	0.087600

## Data Availability

The datasets generated for this study are available upon request to the corresponding author.
